# The effect of antimicrobial treatment on mortality associated with urinary tract disease in mink kits (*Neovison vison*)

**DOI:** 10.1186/s13028-021-00581-3

**Published:** 2021-04-01

**Authors:** Karin Mundbjerg, Peder Elbek Pedersen, Anne Sofie Hammer

**Affiliations:** 1 Department of Veterinary and Animal Sciences, Faculty of Health and Medical Sciences, University of Copenhagen, Ridebanevej 3, 1870 Frederiksberg C, Denmark; 2 LVK Dyrlægerne A/S, Fynsvej 8, 9500 Hobro, Denmark

**Keywords:** Antibiotics, Cystitis, Treatment, Urolithiasis, Veterinary

## Abstract

Mink urinary tract disease (MUTD) often presents as urolithiasis and/or cystitis and is known as an important cause of mortality in mink kits during the early growth season. Antimicrobial flock treatment has been routinely applied as preventive/therapeutic protocol on Danish mink farms with increased mortality associated with MUTD. The therapeutic effect of this treatment strategy has not previously been investigated. In this study, we applied controlled parallel group treatment trials to assess the effect of sulfadiazine/trimethoprim and amoxicillin treatment on mortality associated with MUTD in mink kits. On farm A, eight mink kits were diagnosed with MUTD post mortem in the treatment group (n = 1920, sulfadiazine/trimethoprim treatment: 30 mg/kg, q 24 h, P.O for 5 days) compared to 16 in the untreated control group (n = 1920). No significant difference in mortality associated with MUTD were found between  the treatment and the control group using the Fisher’s exact test (P = 0.15). Treatment group 2 (n = 1920, amoxicillin treatment: 14 mg/kg q 24 h, P.O for 5 days) and treatment group 3 (n = 2088, amoxicillin treatment: 7.5 mg/kg q 24 h, P.O for 5 days) were investigated on farm B. Eight and four mink kits were diagnosed with MUTD post mortem in group 2 and 3, respectively. No difference between occurrence of MUTD were found between the control group and treatment group 2 (P = 0.42) or treatment group 3 (P = 0.75). No significant difference between final body weights or weight gain were found between treatment and control weighing groups on farm A or B. In conclusion, antimicrobial treatment administered in the feed showed no significant effect on weight gain or mortality associated with MUTD on the farms included in this study.

## Findings

Mink urinary tract disease (MUTD) presents as urolithiasis and/or cystitis diagnosed post mortem in mink kits [1–5]. Both bacterial infection and struvite urolithiasis have been suggested as aetiological factors [1, 2, 6, 7]. Recent studies identified *Staphylococcus delphini* Group A as part of the mink skin microbiota and an opportunistic pathogen associated with MUTD [1, 8].

Use of antimicrobial flock treatment to target cystitis [9] and prevent MUTD is common veterinary practice in Denmark when MUTD associated mortality occurs. Considering the potential adverse effects and the lack of knowledge of the pathogenesis of MUTD, this treatment strategy is questionable. The aim of this study was to assess the effect of the currently applied treatment strategy on mink farms experiencing mortality associated with MUTD in mink kits.

Controlled parallel group treatment trials were performed on two Danish mink farms in Jutland during July–September 2018 (farm A) and July–August 2020 (farm B). The clinical trial protocol was approved by the Danish Medical Agency. Kits 2 months of age were included. Mink kits housed on farm A were of fur colour types brown and white and 1920 (n) of each type were included. Animals on farm B were of colour type brown and a total of 5976 was included. Mink kits were housed in traditional sheds with standard cages meeting the requirements of Danish law (one female and male kit in each). Animals were fed a standard mixed feed supplied daily from one local commercial feed kitchen. Farm A used a daily feed additive of ammonium chloride (2–3‰) from end of treatment to trial termination.

The farmers contacted their farm veterinarian because MUTD spontaneously occurred in early July. The farms met the inclusion criteria of the study specified in Table 1. During necropsy (at trial inclusion) bladder swabs (n = 4 from each farm) were collected from mink with lesions compatible with MUTD without visual signs of decomposition. The swaps were submitted to routine culture analysis and antimicrobial susceptibility test.

**Table 1:** Farm mortalities, sampling and necropsy results of MUTD (mink urinary tract disease) during study enrolment

	Average kit mortality before recognition of clinical features^a^ of MUTD		Average kit mortality after recognition of clinical features^a^ of MUTD		Fraction of necropsied kits with MUTD^b^
	Dates^c^	‰ per day	Dates^c^	‰ per day	
Farm A	27/6–29/6 2018	0.3	3/7–5/7 2018	0.6	11^d^/12
Farm B	23/6–27/6 2020	0.3	1/7–5/7 2020	0.7	10^d^/12

Study inclusion criteria: 100% increased kit mortality caused by MUTD (minimum 2/3 of necropsied mink kits diagnosed post mortem with MUTD)

^a^Farmer observing elevated mortality of especially male mink kits

^b^When investigating the cause of elevated mortality in early July

^c^The number of days for calculation of average mortality was doubled from 2018 to 2020 to strengthen the argument of elevated mortality

^d^Bladder swabs (n = 4) were collected for routine microbiological culture analysis and antimicrobial susceptibility test from mink kits with MUTD not showing visual signs of decomposition

Animals were randomly selected for trial groups by row (farm A) or house (farm B). Animal data is presented in Table 2. From each group 42 animals were randomly selected for weighing groups. Weight of the same animal was recorded at initiation and 12 weeks later and weight gain calculated. On farm A, treatment group 1 (n = 1920) were administered one daily dose of 30 mg/kg sulfadiazine/trimethoprim for 5 days. At farm B, treatment group 2 (n = 1920) were administrated a daily dose of 14 mg/kg amoxicillin for 5 days and treatment group 3 (n = 2088) received a daily dose of 7.5 mg/kg amoxicillin for 5 days. Treatment was administered in feed by adding a daily prepared stock solution to the water supply of the feeding machine at one daily feeding. Preparation and administration of the stock solution were supervised by the research group. The protocol included instruction for handling of kits with clinical signs of MUTD (the normal farm practice), but no cases were observed.

**Table 2:** Displaying trail groups at farm A and B including enrolled animals, treatment and weight groups

	Farm A	Farm B
Antimicrobial agent	Sulfadiazine 200 mg/g + trimethoprim 40 mg/g (Trimazin Forte Vet.)	Amoxicillin 697 mg (Octacillin Vet.)
Trail duration	7th of July to 1st of October 2018	9th of July to 1st of September 2020
	*Treatment group 1*	*Treatment group 2*
Treatment specifications	Sulfadiazine/trimethoprim: 30 mg/kg q 24 h, P.O for 5 days	Amoxicillin: 14 mg/kg q 24 h, P.O for 5 days
Mink kits (n)	1920	1920
Color type (n)	Brown (n = 960); White (n = 960)	Brown (n = 1920)
Weight group	42 (n) males (21 brown, 21 white) 42 (n) females (21 brown, 21 white)	42 (n) brown males 42 (n) brown females
		*Treatment group 3*
Treatment specifications		Amoxicillin: 7.5 mg/kg q 24 h, P.O for 5 days
Mink kits (n)		2088
Color type (n)		Brown (n = 2088)
Weight groups		42 (n) brown males 42 (n) brown females
**Control group**		
Treatment specifications	Administered pure water in the same amount as the treatment group	Administered pure water in the same amount as the treatment group
Mink kits (n)	1920	1968
Color type (n)	Brown (n = 960); White (n = 960)	Brown (n = 1968)
Weight groups	42 (n) males (21 brown, 21 white) 42 (n) females (21 brown, 21 white)	42 (n) brown males 42 (n) brown females

Throughout the study period all dead mink kits were collected and stored on − 20 °C until examination. Using previously described procedures [1] urinary organs were evaluated by gross pathological examination and swab samples were collected from the bladder mucosa and/or content of all mink with macroscopic lesions of the urinary tract. Two mink were excluded from sampling because of bladder rupture. Bladder specimens was subjected to microbiological culture and/or MALDI-TOF as previously described [1].

Results of post mortem examinations and statistical analysis of this data are presented in Tables 3 and 4. MUTD was less prevalent in the treatment group 1 (8/1920) compared to the control group (16/1920). There was a significant difference between starting weights of males on farm A. Likewise, there were a significant difference between starting weights of females in group 3 and the control group at farm B. Results of microbiological culture are presented in Table 5. Staphylococci were detected in 70% of mink kits with lesions in farm A and 44% in farm B. Isolates identified as *S. delphini* group A made up 83% of the staphylococci isolated. Antibiotic susceptibility test showed no resistance of cultured staphylococci to sulfadiazine trimethoprim at farm A or amoxicillin at farm B.

**Table 3:** Results of post mortem examination, animal body weights and statistical testing from farm A

Farm A	Treatment group 1	Control group	P-value
Dead kits with urinary tract disease^a^ (n)	8	16	
Remaining mink kits^b^ (n)	1912	1904	0.15^c^
Mortal urinary tract disease prevalence	4.2‰	8.3‰	
	Mean (± SD)		
Start weight females (g)	905 (± 115)	915 (± 107)	0.68^d^
Start weight males (g)	1224 (± 127)	1137 (± 143)	0.004^d^
Final weight females (g)	1951 (± 221)	2010 (± 260)	0.27^d^
Final weight males (g)	3571 (± 353)	3505 (± 371)	0.41^d^
Weight gain females (g)	1046 (± 227)	1095 (± 239)	0.34^d^
Weight gain males (g)	2349 (± 350)	2364 (± 307)	0.84^d^

^a^Gross pathological finding comparable with cystitis, pyelonephritis and/or urolithiasis

^b^Included mink kits not diagnosed with urinary tract disease

^c^Fisher’s exact test

^d^Welch two-sample t-test

**Table 4:** Results of post mortem examination, animal body weights and statistical testing from farm B

Farm B	Treatment group 2 (T2)	Treatment group 3 (T3)	Control group (C)	P-value		
				T2 vs. C	T3 vs. C	T2 vs. T3
Dead kits with urinary tract disease^a^ (n)	8	4	5			
Remaining mink kits^b^ (n)	1912	2084	1963	0.42^c^	0.75^c^	0.25^c^
Mortal urinary tract disease prevalence	4.2‰	1.9‰	2.5‰			
	Mean (± SD)					
Start weight females (g)	1144 (± 97)	1126 (± 135)	1188 (± 129)	0.10^d^	0.02^d^	0.52^d^
Start weight males (g)	1520 (± 256)	1509 (± 202)	1522 (± 224)	0.97^d^	0.80 ^d^	0.83^d^
Final weight females (g)	1288 (± 201)	1225 (± 260)	1258 (± 207)	0.81^d^	0.12^d^	0.18^d^
Final weight males (g)	4206 (± 430)	4140 (± 470)	4130 (± 538)	0.48^d^	0.92^d^	0.54^d^
Weight gain females (g)	1521 (± 235)	1436 (± 343)	1538 (± 268)	0.54^d^	0.52^d^	0.21^d^
Weight gain males (g)	2676 (± 320)	2631 (± 393)	2608 (± 462)	0.44^d^	0.79^d^	0.61^d^

^a^Gross pathological finding comparable with cystitis, pyelonephritis and/or urolithiasis

^b^Included mink kits not diagnosed with urinary tract disease

^c^Fisher’s exact test

^d^Pairwise t-test

**Table 5:** Microbial findings of mink bladder specimens sampled post mortem from farm A (n = 23) and B (n = 16)

Microbial culture findings of bladder specimens^a^		
	Farm A	Farm B
*Staphylococcus delphini* group A (n)	15^b^	4^b^
*Staphylococcus* spp. (n)		3^c^
*Proteus* sp (n)	2^b^	3^c^
*Enterococcus faecalis* (n)	2^b^	1^b^
*Escherichia coli* (n)	2^b^	2^b^
*Staphylococcus aureus* (n)	1^b^	
*Morganella morganii* (n)		1^b^
Sterile (n)	2^c^	5^c^

^a^Sampled from mink kits with gross pathological findings of the urinary tract

^b^Final identification by MALDI-TOF

^c^Identification by culturing

No significant difference was found between mortality associated with MUTD in the treatment and the control groups, by means of the Fisher’s exact test at a 5% significance level (results presented in Table 4). MUTD associated mortality is mainly seen in male mink kits [1, 2, 5]. It remains unknown if females may be subject to subclinical disease associated with MUTD. Also, because the male and female kits share cages and feed [10], the females are routinely included in the treatment and may be affected by this. Therefor females were also included in the study.

**Fig. 1:**
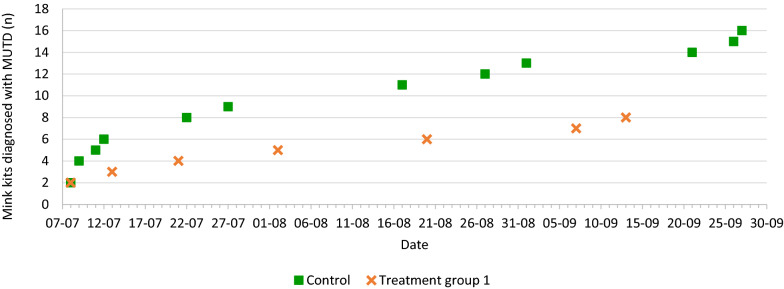
Cumulative number of mink diagnosed with mink urinary tract disease (MUTD) at post mortem according to date. Farm A

**Fig. 2:**
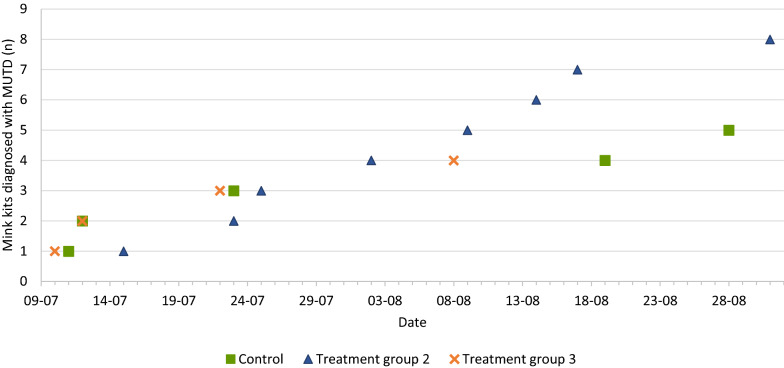
Cumulative number of mink diagnosed with mink urinary tract disease (MUTD) at post mortem according to date. Farm B

The antimicrobial flock treatment initiated after diagnosis of MUTD did not significantly reduce mortality associated with MUTD during the growth period. As illustrated in Figs. 1 and 2, mortality associated with MUTD occurred throughout the investigation period with no obvious culmination of disease. This result may have been affected by the relatively low post mortem prevalence of MUTD in the groups (1.9–8.3‰). Both farms presented with mortality associated with MUTD (0.6 and 0.7‰/day) at the time of inclusion. As a rule of thumb, kit mortality after weaning should not exceed 1‰ per week (0.14‰/day).

Both urolithiasis (56% and 65%) and infection with staphylococci (44% and 70%) were frequent findings. This is in agreement with previous studies investigating MUTD [1, 2, 4]. Urethral obstruction by purulent exudate or uroliths was reported as cause of death in MUTD [1, 2]. It is possible that a combination of antimicrobials and ammonium chloride can lower mortality more efficiently than antimicrobial treatment alone by reducing crystals/uroliths as well as bacterial burden. Similarly, recommendations for prevention of struvite urolithiasis in dogs include antimicrobial treatment in combination with dietary supplements aiming to dissolve uroliths [11–13].

One recent study reported mink of colour type black being predisposed to MUTD compared to brown mink [5]. In recent years numbers of black mink on Danish farms have decreased and there was no black mink on farms included in this study.

Sulfadiazine/trimethoprim and amoxicillin are first choice therapy for uncomplicated urinary tract infections in dogs and cats [14] and also used for treatment of MUTD, though no manufacturer recommendations for use in mink is available. The dose applied in treatment 1 was sufficient to treat *S. delphini* group A and *E. coli* mink infections [15]. The low dose of amoxicillin applied on farm B was included because a novel study suggest it is sufficient for *S. delphini* group A [16]. Long time storage of mink feed mixed with drugs may affect drug concentration [17]. It may take mink kits several hours to ingest all feed and there might be reduction in concentration through this period. Both antimicrobials applied are eliminated through urine which favors high antimicrobial concentration in the bladder [18, 19].

Weight gain in mink kits can be considered an indicator for health and thriftiness. In this study, there were no significant differences between groups when comparing final weights or average weight gain. Thus indicating, that the applied treatment did not affect general health and growth.

Isolates submitted for antimicrobial susceptibility testing were susceptible to the applied antimicrobial drugs, however, only few isolates were tested (n = 4 from each farm). We cannot rule out, that antimicrobial resistance may have contributed to the lack of treatment effect. Bacterial isolates from Danish mink have shown ampicillin resistance in up to 82.3% of *E. coli* isolates [9, 20]. There are no reports of ampicillin resistance in *S. delphini* group, though penicillin resistance were reported in 47% of isolates in a previous study [20]. *S. delphini* group A isolates have been reported to be susceptible to sulfadiazine/trimethoprim [20].

Prevalence of MUTD diagnosed post mortem ranged between 1.9 and 8.3‰ in study groups, which is consistent with previously reported mortality prevalences of MUTD on Danish mink farms [1]. It is always important to be aware of the potential adverse effects of the exposure of healthy mink to antimicrobials when applying flock treatment. This is emphasized by the low prevalence of MUTD recorded by post mortem examination in this and previous studies (implying that a larger proportion of clinically healthy mink is included in the treatment) and reports of frequent detection of antimicrobial resistance in bacterial mink pathogens [9, 20].

In conclusion, antimicrobial treatment had no significant effect on weight gain or mortality associated with MUTD in this study. While flock treatment of farms with MUTD is currently commonly applied, our results do not support this practice. More research is needed to ensure prudent and efficient use of antimicrobials and especially the use of flock treatment, which accounts for most of the antibiotics used for production animals. The results emphasize the need for improved and documented protocols for the prevention of MUTD.

## Data Availability

The datasets used and/or analyzed during the current study are available from the corresponding author on reasonable request.
